# Typhus Fever

**Published:** 1842-08-06

**Authors:** 


### CLINICAL REPORTS.

### *Blockley Hospital—Service of W. W. Gerhard, M. D.*

Reported by M. W. Wilson, M. D., Resident Physician.

### *Typhus Fever.*

This disease, when it occurred in young men, assumed a very mild form. It passed through its stages in about twelve to fifteen days, and the patients convalesced. This was not only true in regard to the patients treated within the last seven weeks; but for the last seven or eight months not a patient has died with this disease under the age of 60 years; and we believe that most of the cases of typhus that occurred in individuals above that age proved fatal.

There was but one case treated in these wards, within the time specified in this report, above 60 years of age. This man was upwards of 80. He was brought to the hospital from prison, and was almost speechless, from extreme feebleness, when he entered the ward. The characteristic eruption over the abdomen, and the peculiar drunken expression of the eyes, together with the general debility, and the absence of any local affection, left no doubt of the disease. He died three days after he came to the hospital, and on examination, the blood was found in a fluid state, with no visceral affection except a little bright red injection of the mucous coat of the stomach.

The intestinal follicles, as is usual in this variety, were healthy.

The other cases occurred in young vigorous men, and they all terminated favourably. There were three cases in all. One case will serve to illustrate the disease as it presented itself this season.

J. G., ætat. 24, an Englishman, entered the ward May the 20th. He landed from the Swatara about three days previous to his entrance. He was taken ill about three weeks previous, while on the vessel, with headache and "sea sickness." When the proper fever was developed it was impossible to say. There was considerable sickness on board at that time. This is all the history that we could get from him.

When he entered the ward the symptoms were as follows: Slight cephalalgia; skin dry and harsh; conjunctiva injected; eyes suffused: expression of drunkenness; tongue yellowish and moist; almost complete anorexia. Pulse 112, small and feeble; respiration 24, regular; bowels constipated for two days. The abdomen was covered with a very slight eruption, apparently just commencing. No deafness; no delirium; no subsultus tendinum, and no constant pain, but extreme prostration. He was ordered a warm pediluvium, and a dose of oil.

21st. Eruption more distinct over abdomen; tongue coated and dry; skin hot; pulse 116, feeble; complete anorexia. Bowels opened gently with the oil. He was directed to be sponged with tepid water, and to have a warm pediluvium night and morning; to take four ounces of wine a day.

℞. Infus. Serpentar. ℥ii. q. 4 h.

℞. Liq. Ammon. Acet. ℥ss. q. 2 h.

22d. Eruption more developed; slight cephalalgia; insomnolence; pulse

120, feeble; skin warm and dry; thirst. Directed a weak solution of citric acid, and the other treatment to be continued.

23d. Intellect dull; answers slow; memory almost gone; expression stupid; eyelids slightly tumid; skin dry; pulse easily compressed, 112. Eruption well developed; it now differs in size from points scarcely perceptible to spots the sixth of an inch in diameter, which nearly disappear under pressure. No sudamina; extreme cutaneous sensitiveness. He was directed a dose of oil, and six dry cups to the nucha, and the other treatment to be continued.

24th. Pulse 84, feeble; less cutaneous sensitiveness; no pain; slept a little. Treatment continued.

25th. Skin cool; pulse small and feeble, 78; eruption fading; tongue clammy; some thirst. Treatment continued.

26th. Sleep disturbed; pulse 104, feeble.

27th. Countenance much improved; answers questions readily; appetite returning; skin cool; pulse 80, small, rather feeble; eruption disappearing; cutaneous sensibility less. He was now considered convalescent, and from this time rapidly improved. The eruption was gone on the 30th.

